# Editorial COVID-19 and Thrombosis 2023: New Waves of SARS-CoV-2 Infection, Triage Organization in Emergency Department and the Association of VOCs/VOI with Pulmonary Embolism

**DOI:** 10.3390/v14112453

**Published:** 2022-11-05

**Authors:** Ciro di Gennaro, Mariano Galdiero, Giovanna Scherillo, Stefano Parlamento, Maria Rita Poggiano, Claudia Arturo, Antonio Vasta, Beniamino Giordano, Viviana Pisano, Antonio Lobasso, Giuseppe Camporese, Pierpaolo Di Micco

**Affiliations:** 1 Department of Medicine PO A Rizzoli, ASL Napoli 2 Nord, Lacco Ameno, 80076 Naples, Italy; 2 UO Internal Medicine, Azienda Ospedaliera di Padova, Via Giustiniani 2, 35128 Padova, Italy

Nearly two years ago, the SARS-CoV-2 outbreak began, and our lives have changed significantly since then. The increasing the number of submissions and articles to the scientific literature from around the world has contributed to the knowledge of this virus and its related infectious diseases. In actual fact, the pandemic is still ongoing, although with different clinical approaches because of a series of new insights. In order to fight the pandemic, aside from personal precautions (e.g., face mask, access to public areas reduced to a decreased number of people at one time, the frequent cleaning of hands and the climatic variation and circulation of the virus), the progression of the vaccination campaign has reduced the number of infected patients with severe lung failure month by month and as well as reducing morbidity, mortality and the hospitalization rate for COVID-19. However, there are still several extra issues regarding the management of subjects affected by COVID-19, in particular when hospitalization is required, and this clinical trend has also changed during the submission period of this Special Issue, reflecting the different clinical presentations of COVID-19.

The first waves of COVID-19 were characterized by the association of lung failure with pulmonary embolism, bacterial/fungal over-infection and the increase in inflammatory markers that acted as prognostic markers and also as the target of pharmacological treatment (e.g., IL-6). In these first phases of the pandemic, the useful role of low molecular weight heparin, such as enoxaparin, has been underlined in several reports, and Imbalzano et al. [1] undertook the analysis of patients with an increased thrombotic risk of developing VTE as oncological patients as far as the increased risk of COVID-19 for patients with comorbidities, such as cardiovascular diseases [2,3]. As for other viruses, SARS-CoV-2 quickly began its cycle of genome mutation that induced its prolonged survival in the face of the immunization of the general population. For this reason, several viral variants have been identified in the last two years: B.1.1.7 (Alpha), B.1.351 (Beta), P.1 (Gamma), B.1.617.2 (Delta), B.1.427, B.1.429 (Epsilon), P.2 (Zeta), B.1.525 (Eta), P.3 (Theta), B.1.526 (Iota), B.1.617.1 (Kappa) and B.1.1.529 (Omicron) and its subvariants [4,5], which are the protagonists of the prolongation of the SARS-CoV-2 outbreak and are named variants of concern (VOCs) or variants of interest (VOI), according to WHO and the European Centre for diseases prevention and control (ECDPC). From a clinical point of view, VOCs/VOI may also be responsible for the late identification of SARS-CoV-2 infections through nasopharyngeal swabs (NPS). Therefore, the identification of SARS-CoV-2 infection is necessary not only with NPS but also through the research of other biological fluids or tissues, such as bronchoalveolar lavage or urine [6]. Furthermore, in particular for hospital workers, the high chance of contracting COVID-19 or a COVID-19-like syndrome in the presence of suspected signs and symptoms even when repeated NPS did not reveal the presence of SARS-CoV-2 has been underlined by Di Micco et al. [6].

Furthermore, despite the vaccination campaign against SARS-CoV-2, there are several subcategories of patients that are still at risk of developing severe infection by SARS-CoV-2, such as unvaccinated individuals, patients with immunodepression from any cause (i.e., related to underling disease or to chronic treatment with drugs that may induce immunodepression) or patients with low immunological and clinical response to the SARS-CoV-2 vaccination [7,8].

For this reason, the management of a suspected infection SARS-CoV-2 for the triage system is very difficult and “grey areas” may be necessary in order to identify different subgroups of infected patients with SARS-CoV-2, in particular in emergency departments. Grey areas help physicians to distinguish patients at high risk of developing severe COVID-19 (e.g., unvaccinated individuals or non-responding patients to the SARS-CoV-2 vaccination) from those with less severe respiratory infection by viral VOCs but with an associated other medical illness that may require hospitalization (e.g., cardiological illness, neurological illness, surgical/orthopaedic diseases or pregnancy-associated diseases). Furthermore, the presence of a grey area may also be useful to identify patients with a high probability of presenting symptoms and signs of respiratory infection by SARS-CoV-2 VOCs but which may be negative to NPS.

In our clinical experience, the identification of grey areas in emergency departments has helped physicians to escape mistaking treatment priority and treatment location, as reported in our Figure 1. The previously suggested subdivision in patients with respiratory and non-respiratory symptoms in another suggested flow chart [9] should be further subdivided based on the possibility of a relapse of infection or other clinical troubles (e.g., not associated with lung injuries).

Furthermore, the association between venous thromboembolism and COVID-19 decreased after the first waves and after the vaccination campaign and the appearance of new VOCs and VOI of SARS-CoV-2. The previous waves of COVID-19, in fact, demonstrated a strong association between COVID-19 and pulmonary embolism, in particular in patients intensive care unit and those in subintensive care units with associated increased morbidity and mortality [10,11]. Intriguingly, during the last waves of COVID-19, the rate of PE seems to have decreased [12], according to several conditions as the routine thromboprophylaxis of infected patients, reduced the length of the hospitalization of infected patients and reduced the virulence of VOCs and VOI also associated with previous SARS-CoV-2 vaccination. However, non-responders to vaccination, unvaccinated individuals and frail patients with co-existing immunological defects (e.g., related to underling diseases or the chronic use of specific drugs) may be affected by severe COVID-19 and its typical complications, such as overlapping bacterial or fungal infection or associated pulmonary embolism. For this reason, clinical scores and biomarkers to identify PE in COVID-19 may be always used. In particular, the use of d-dimer is always suggested and, in our experience, a doubling d-dimer [13] in patients admitted to the emergency room for COVID-19 should be always associated with the use of an objective test to detect PE associated with COVID-19 (Figure 2).

In conclusion, we can assert that during the pandemic we learnt of and improved several clinical medical issues. Therefore, the management of patients infected by SARS-CoV-2, regardless of the type of VOCs or the referred acute symptoms when admitted to the emergency department, is extremely topical and useful in order to optimize the quality of medical assistance.

## Figures and Tables

**Figure 1:**
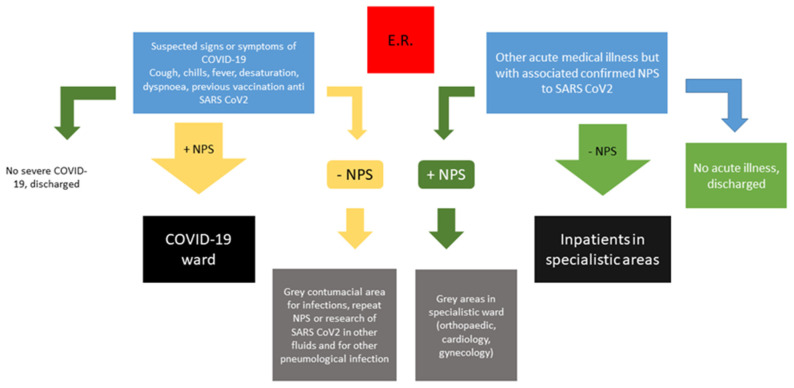
Updated flow chart for managing patients with confirmed or suspected infection by SARS-CoV-2, updated for summer/autumn 2022.

**Figure 2:**
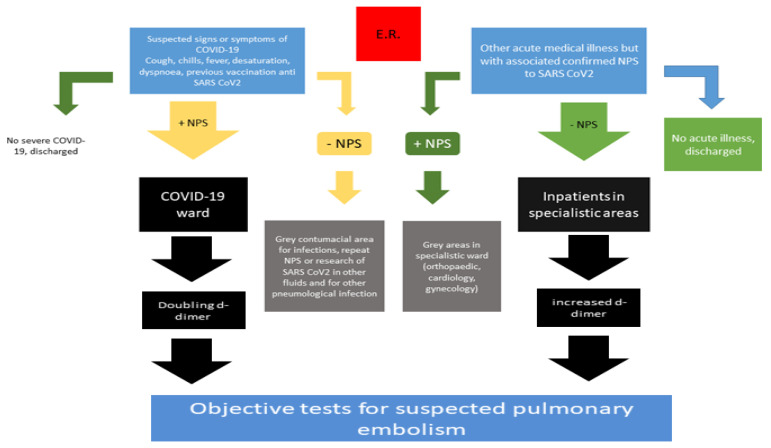
Updated flow chart for managing patients with confirmed COVID-19 and suspected pulmonary embolism, updated to summer/autumn 2022.

## Data Availability

Not applicable.
